# Cactus (*Opuntia humifusa*) water extract ameliorates loperamide-induced constipation in rats

**DOI:** 10.1186/s12906-016-1552-8

**Published:** 2017-01-17

**Authors:** Sung Hee Han, Kyungmi Park, Eun Young Kim, So Hyun Ahn, Hyun-Sun Lee, Hyung Joo Suh

**Affiliations:** 1 BK21Plus, College of Health Science, Korea University, Seoul, 02841 Republic of Korea; 2 Regulatory Affairs & Product Compliance Korea and Philippines, Herbalife Korea, Seoul, 06052 Republic of Korea; 3 Department of Food and Nutrition, Korea University, Seoul, 02841 Republic of Korea; 4 Agency for Korea National Food Cluster, Iksan, 54622 Republic of Korea; 5 Department of Public Health Sciences, Graduate School, Korea University, Seoul, 02841 Republic of Korea

**Keywords:** Cheonnyuncho, *Opuntia humifusa*, Constipation, Loperamide, Gastrointestinal transit

## Abstract

**Background:**

Korean cactus Cheonnyuncho (*Opuntia humifusa*) is rich in pectin, phenols, flavonoids, and minerals such as calcium and phosphorus. Some Koreans drink Cheonnyuncho juice prepared by grinding Cheonnyuncho with water. Cheonnyuncho is well known for its functional properties and antioxidant effects, but its effect on constipation has not been sufficiently studied.

**Methods:**

Loperamide (2 mg/kg) was injected subcutaneously to induce constipation in rats. The animals were divided into four groups: a normal group (NOR), constipation control group (CON), and two constipation groups receiving the Cheonnyuncho extract (CE) at two different concentrations in drinking water, 3% (L-CE group) and 6% (H-CE group), for 25 days.

**Results:**

The fecal pellet numbers of NOR and L-CE were significantly increased from 35.67 ± 2.09 (CON) to 50.60 ± 1.38 and 46.50 ± 2.91 after loperamide treatment, respectively (*p* < 0.05). The water content of fecal excretions was significantly enhanced in only the L-CE group (33.05 ± 0.49%) compared to control (23.38 ± 1.26%) (*p* < 0.05) after loperamide treatment. The oral intake of CE (L-CE and H-CE groups) significantly increased levels of the intestinal transit ratio (45.25 ± 1.86% and 41.05 ± 2.47%, respectively) compared to the CON group (32.15 ± 2.05%) (*p* < 0.05). Treatment with the low concentration of CE significantly increased fecal levels of acetic, propionic, butyric, and valeric acids, as well as the total short-chain fatty acid (SCFA) concentration. Histological analyses revealed that the thickness of the distal colon also increased in the CE-treated groups in a dose-dependent manner.

**Conclusions:**

Constipation decreased when CE was fed to the rats. In particular, the fecal pellet number and water content, as well as histological parameters such as distal colon thickness, improved. The CE treatment also increased the fecal SCFA content. These results show that the extract of Cheonnyuncho (*O. humifusa*) alleviated the symptoms of loperamide-induced constipation.

## Background

Constipation refers to bowel movements that are infrequent or hard to pass and is a common cause of painful defecation. Severe constipation includes a failure to pass stools or gas and fecal impaction, which can progress to bowel obstruction and become life threatening. Constipation is a symptom with many causes, including mechanical and functional ones [1]. Causes of colonic slow-transit constipation include diet, hormonal disorders such as hypothyroidism, side effects of medications, and infrequently, heavy metal toxicity [2]. Because constipation is a symptom, not a disease, effective treatment of constipation may first require determining the cause. Treatments include changes in dietary habits, laxatives, enemas, biofeedback, and in particular situations, surgery. Constipation is common, and its incidence in the general population varies from 2 to 30% [1, 3, 4].

The food and pharmaceutical industries have expressed an increasing interest in functional materials with biological activities [5]. Naturally derived materials are often as effective in disease control as chemically synthesized materials, and consumer preference is higher for the former because of their low toxicity and fewer side effects [5].

Medicinal properties of *Opuntia humifusa* have been explored in recent years [6, 7]. It has been reported that extracts of *O. humifusa* exhibit anti-inflammatory and radical-scavenging activities by suppressing the expression of inducible nitric oxide synthase, as well as cytokines including interleukin (IL)-6 and IL-1β, in lipopolysaccharide-treated RAW 264.7 cells [8]. Hahm et al. [9] reported that oral administration of *O. humifusa* showed antidiabetic activity owing to a decrease in blood glucose levels in streptozotocin-induced diabetic rats, caused by an increase in the relative beta-cell volume in the pancreas [9]. *O. humifusa* is also a high-fiber source and is characterized by mucilage production. Mucilage, which is a complex of carbohydrates, is classified as a dietary fiber. Its main component is pectin (48.5%), but mucilage also contains arabinose, galactose, galacturonic acid, and rhamnose [10]. *O. humifusa* is a good source of dietary fiber, and when added to food as a supplement, can provide beneficial health effects and prevent colonic diseases. Oral intake of the fruit or stem of *O. humifusa* on an empty stomach has been shown to be effective in treating constipation and urination problems, increasing intestinal movement, and improving appetite [11]. However, scientific evidence of the correlation between *O. humifusa* intake and constipation improvement has not been presented to date. Therefore, it is worth investigating the laxative effects of an extract of the cactus in an animal model of constipation to verify its mechanism of action under physiological conditions. In this study, we investigated the effect of an extract of the cactus on loperamide-induced constipation in rats.

## Methods

### Preparation of Cheonnyuncho water extract

Cheonnyuncho grown in Sejong, Korea, was harvested in October 2014, washed with water, which was then removed with kitchen paper towels, and stored in a refrigerator. The identity of the plant was ascertained morphologically by Prof. K. S. Shin of Kyonggi University, Suwon, Korea. A voucher specimen (KUCHS-2012008) was deposited at the College of Health Sciences, Korea University, Seoul, Korea. The Cheonnyuncho extract (CE) was prepared as described in our previous study [12]. Briefly, cactus cladodes (10.0 kg) were homogenized with 50.0 L of water. Then, the suspension was hydrolyzed with 250 mL of an enzyme solution [pectinase (2400 U/g) and cellulase (1200 U/g), 1:3 (v/v)] at 45 °C for 6 h. The reaction was stopped by heating to 95 °C for 20 min, followed by cooling to room temperature (23 ± 0.5 °C) The resulting crude extract was filtered and lyophilized to a dry powder.

### Cheonnyuncho dietary fiber and mucilage analysis

The amounts of soluble dietary fiber (SDF), insoluble dietary fiber (IDF), and total dietary fiber (TDF) were determined by the gravimetric enzymatic method as previously described by Prosky et al. [13]. For the extraction of mucilage, nopal pads were homogenized with water at a ratio of 1:5 by using an Ultra Turrax model T25 basic homogenizer (IKA Works, Wilmington, NC, USA), and then the mixture was filtered through a fine cloth. The resulting solution was concentrated in vacuum and poured into ethanol (1:5, v/v). The precipitate was separated by centrifugation (5000 g, 1 h), dissolved in distilled water, and freeze-dried.

### Experimental animal groups

Male Sprague–Dawley rats (*n* = 24), with an average weight of 170 g, were purchased from Deahan Biolink (Seoul, South Korea) and maintained at animal facilities of Korea University (Seoul, South Korea) after adaptation for 1 week. Each rat was kept in a specific, pathogen-free room at 24 ± 0.5 °C with relative humidity of 50–55% under 12-h day/night cycles. A standard maintenance diet (Purina rodent chow) and water with/without CE were freely provided in the cage for 25 days. All experiments were performed according to the protocols approved by the Institutional Animal Care and Use Committee at Korea University (KUIACUC-2013-198).

The animals were randomly divided into a normal group (NOR) and three experimental groups, a control group (CON), L-CE group (3% CE added to drinking water), and H-CE group (6% CE added to drinking water). There were six animals assigned to each group. The animals in the NOR and CON groups received only water, without CE. In the experimental animals, constipation was induced by loperamide hydrochloride (Sigma Chemical Co., St. Louis, MO, USA) injection after CE intake (L-CE and H-CE groups) or water intake (CON group). The rats were injected with loperamide (2 mg/kg of body weight) in a saline solution subcutaneously twice per day, at 09:00 and 18:00, for 7 days.

### Body weight gain, food intake, and food efficiency ratio

We measured the body weight gain and food intake before inducing constipation with loperamide on week 1 of the experiment and then again after inducing constipation. The food efficiency ratio was calculated by dividing the dietary intake by weight gain during the same period.

### Wet and dry weight of fecal pellets

The wet and dry weights of fecal pellets of the animals were measured. Fecal samples were collected before the induction of constipation four times every day for 1 week after the experiment started and once every day during the collection period of constipation [14]. We calculated changes in the wet weight of fecal pellets and water content of fecal pellets. The water content was determined by drying fecal pellets at 70 °C for 24 h in an oven and calculating the difference between the weight before and after drying.

### Serum lipids

To analyze the content of triglycerides, total cholesterol, and high-density lipoprotein (HDL) cholesterol in the serum, the experimental animals were anesthetized on the last day of the experiment, and blood was collected from the abdominal vein via laparotomy. Serum was obtained from the collected blood by centrifugation (2000 g, 10 min) and analyzed by using an automated serum analyzer (Dri-chem 3500i, Fujifilm, Tokyo, Japan). Serum triglycerides, total cholesterol, and HDL cholesterol were measured using the TG-0024, TCHO-0222, and HDL-0233 kits (Fujifilm), respectively.

### Gastrointestinal transit ratio

The movement rate of CE in the intestinal tract was determined by the method described by Yu et al. [15] with some modifications. The CE powder was dissolved in drinking water before the oral challenge with 1 mL of charcoal meal (80 mg of charcoal). After 30 min, the rats were killed, and the intestinal tract was excised. The distance traveled by the charcoal meal from the pylorus was measured and expressed as a percentage of the total length of the small intestine from the gastro-pyloric junction to the ileocecal junction as follows:

$$ \mathrm{T}\left(\%\right)=\mathrm{B}/\mathrm{A}\times 100 $$

where T is the intestinal tract transit ratio, A is the total length of the intestinal tract, and B is the moving distance of the most distal end portion of the charcoal.

### Concentration of short-chain fatty acids

In order to measure the amount of short-chain fatty acids (SCFAs), feces were collected. One gram of feces was extracted with 5 mL of methanol in a conical tube, and the extract was stored at −60 °C until use. The extracted sample was filtered through a 0.45-μm filter (Millipore, MA, USA). The filtrate was analyzed using a DB-FFAP 123–3253 column (50 mm × 0.32 mm i.d., coated with 0.50-μm film; J&W Scientific, Agilent Technologies, Inc., USA) and a gas chromatography system (YL-6100, Yong-Lin Co., Seoul, Korea) equipped with a flame ionization detector and autosampler (HT 300, Toung-Lin Co., Seoul, Korea). The injection amount of the sample was 1 μL, the inlet and detector temperatures were 200 and 240 °C, respectively, and the analysis conditions were as described by Demigne et al. [16]. The content of acetic, propionic, butyric, and valeric acids was obtained from a calibration curve created using a respective standard reagent.

### Histology of the distal colon

Intestinal tissue was embedded in paraffin, and tissue sections (5 μm) were deparaffinized with xylene. After deparaffinization, the tissues were treated with ethanol for 5 min. Then, the samples were stained with Alcian blue for 30 min and rinsed with water. After rinsing, the tissues were stained with a nuclear fast red solution for 30 s and re-dehydrated with ethanol. Then, the tissues were dehydrated, washed, and sealed using xylene. Alcian blue-positive intestinal mucosa cells were observed with an optical microscope (Axiovert S100, Zeiss, Bovenden, Germany).

### Statistical analysis

All statistical analyses were performed using the Statistical Package for Social Sciences, version 12.0 (SPSS, Inc., Chicago, IL, USA). Analysis of variance was performed, and differences among samples were determined using Duncan’s multiple range tests at a significance level of *p* < 0.05. All data were at the 95% significance level and are reported as mean ± standard deviation (SD) values.

## Results

### Dietary fiber and mucilage content of Cheonnyuncho

Compared with other plants, *Opuntia* spp. cladodes exhibit very strong physical properties, such as water-holding capacity, swelling, and viscosity. These are attributable to starchy polysaccharides and IDF, which exhibit poor physical properties in hydration treatments [17, 18]. Fiber is an essential component in a healthy diet and is needed to maintain optimal health. However, dietary fiber intake is often insufficient in all populations [19, 20]. For this reason, fiber is a widely used ingredient in the functional food industry.

An important difference was observed in this study in the TDF content, which was quite high in the products. The major component of the two samples studied was dietary fiber. These results are summarized in Table 1. The SDF, IDF, and TDF contents of Cheonnyuncho were 18.92, 39.15, and 58.07 g/100 g, respectively. After the extract preparation, the SDF, IDF, and TDF contents of CE were 4.71, 14.74, and 19.45 g/100 g, respectively. The results showed a higher SDF content than that reported previously for *O. ficus-indica* cladodes, in which the SDF, IDF, and TDF contents were 5.7–13.0, 30.4–44.1, and 41.8–51.2 g/100 g, respectively [17].

**Table 1:** Total, insoluble, and soluble dietary fiber and mucilage contents of Cheonnyuncho (g/100 g)

Sample	Total dietary fiber (TDF)^a^	Insoluble dietary fiber (IDF)	Soluble dietary fiber (SDF)	Mucilage
Cheonnyuncho	58.07 ± 4.48	39.15 ± 0.52	18.92 ± 3.96	64.74 ± 4.67
Cheonnyuncho extract	19.45 ± 2.00	14.74 ± 0.83	4.71 ± 1.17	46.12 ± 2.61

^a^Calculated by using the following equation: TDF = IDF + SDF

### Number of fecal pellets and fecal water content in experimental animals

**Fig. 1:**
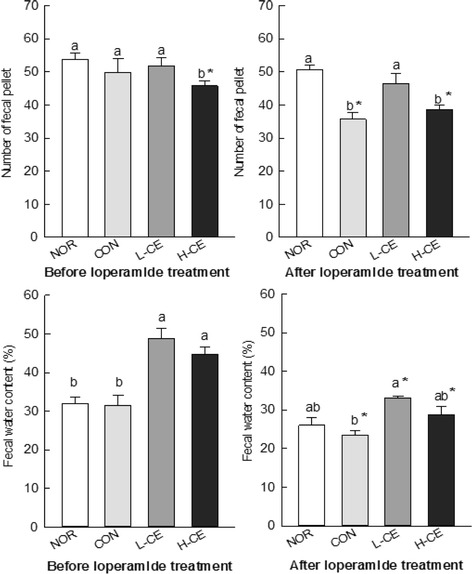
Effects of the CE on the number of fecal pellets (**a**) and fecal water content (**b**) before and after loperamide-induced constipation in a rat model. *Bars* represent the mean ± SD (*n* = 6). Different letters on each graph indicate significant differences (*p* < 0.05) among the groups. The *asterisk* indicates significant differences between respective groups before and after loperamide treatment, based on the Tukey’s test. CE, Cheonnyuncho extract; NOR, normal group; CON, constipation control group; L-CE, constipation plus low-dose CE group (3% CE added to drinking water); H-CE, constipation plus high-dose CE group (6% CE added to drinking water)

Constipation was assessed by the number of fecal pellets and fecal water content to determine the effects of CE on the loperamide-induced rat model. The numbers of fecal pellets did not significantly differ in the NOR, CON, and experimental groups (L-CE and H-CE) before loperamide-induced constipation. Reduced fecal excretion in the loperamide-administered rats compared to that in the normal rats confirmed that loperamide induced constipation (Fig. 1). The fecal pellet numbers and fecal water content in the NOR group were not significantly different from those in the CON group before loperamide treatment. However, the fecal pellet numbers in the NOR (50.60 ± 1.38) and L-CE (46.50 ± 2.91) groups were significantly higher than those in the CON group (35.67 ± 2.09) after loperamide treatment (*p* < 0.05). The water content of fecal excretions was significantly enhanced (*p* < 0.05) only in the L-CE group (33.05%) compared to that in the CON group (23.38%) after loperamide treatment.

### Effect of CE on intestinal transit ratio

**Fig. 2:**
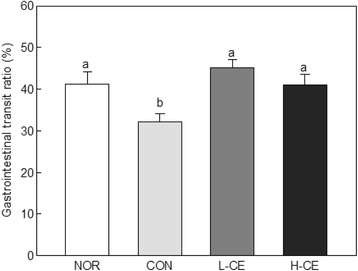
Effects of the CE on the gastrointestinal transit ratio in normal and loperamide-treated rats. *Bars* represent the mean ± SD (*n* = 6). Different letters indicate significant differences (*p* < 0.05) among the groups, based on the Tukey’s test. CE, Cheonnyuncho extract; NOR, normal group; CON, constipation control group; L-CE, constipation plus low-dose CE group (3% CE added to drinking water); H-CE, constipation plus high-dose CE group (6% CE added to drinking water)

The results of gastrointestinal transit ratio are shown in Fig. 2. Compared with that in the NOR group (41.18%), the intestinal transit ratio in the CON group, which was only administered with loperamide, significantly decreased to 32.15% (*p* < 0.05). These results show that the moving rate decreased upon the use of loperamide to induce constipation. The oral intake of CE (L-CE and H-CE groups) was shown to significantly improve the intestinal transit ratio in the rats with loperamide-induced constipation (*p* < 0.05). There was no difference between the two groups with the different intake concentrations of CE. This result indicates that the constipation induced by loperamide treatment was alleviated by the intake of CE. Both CE and cactus stems have been used to treat constipation [21].

### Triacylglycerol, total cholesterol, and HDL cholesterol

The effects of CE on the triacylglycerol, total cholesterol, and HDL cholesterol levels are shown in Table 2. Triglyceride, total cholesterol, and HDL cholesterol levels were not significantly different among the L-CE, H-CE, and CON groups. In the L-CE and H-CE groups, HDL cholesterol levels increased compared with those in the CON and NOR groups, but there were no significant differences. The HDL cholesterol level had a tendency to increase with the L-CE and H-CE treatments, and there are studies reporting similar results [7, 22].

**Table 2:** Effects of CE on serum triacylglycerol, total cholesterol, and HDL cholesterol levels in the loperamide-induced constipation rat model

Lipid	Groups			
	NOR	CON	L-CE	H-CE
Triacylglycerol (mg/dL)	78.75 ± 11.90^ns^	63.00 ± 8.57	61.33 ± 20.96	78.33 ± 26.78
Total cholesterol (mg/dL)	71.20 ± 5.52^ns^	75.53 ± 9.77	68.09 ± 9.77	67.34 ± 3.00
HDL cholesterol (mg/dL)	24.47 ± 2.23^ns^	24.61 ± 1.70	24.31 ± 2.38	27.32 ± 3.12

Mean ± SD (*n* = 6). *ns* not significantly different among the groups, *NOR* normal group, *CON* constipation control group, *L-CE* constipation plus low-dose CE group (3% CE added to drinking water), H-CE constipation plus high-dose CE group (6% CE added to drinking water), *CE* Cheonnyuncho extract, *HDL* high-density lipoprotein

### Concentrations of SCFAs

**Fig. 3:**
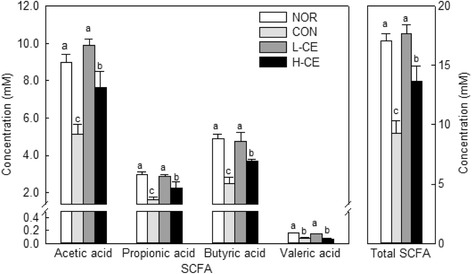
Concentrations of acetic, butyric, propionic, and valeric acids and the total concentrations of SCFAs in feces of untreated and loperamide-treated rats. *Bars* represent the mean ± SD (*n* = 6). Different letters indicate significant differences (*p* < 0.05) among the groups, based on the Tukey’s test. SCFA, short-chain fatty acid; NOR, normal group; CON, constipation control group; L-CE, constipation plus low-dose CE group (3% CE added to drinking water); H-CE, constipation plus high-dose CE group (6% CE added to drinking water)

It has been reported that SCFAs, which are the main final products of intestinal bacterial fermentation of polysaccharides, have a laxative effect by affecting the enteral mucosa [23]. The fecal concentrations of acetic, butyric, propionic, and valeric acids and the total concentrations of SCFAs are shown in Fig. 3. The contents of acetic, propionic, butyric, and valeric acids in the L-CE group were significantly different from those in the CON group, indicating that constipation alters the SCFA chemical composition. CE fermentation by microflora leads to the production of SCFAs, which lower colonic pH. Therefore, the increase in the contents of acetic, propionic, butyric, and valeric acids in the fecal samples can be attributable to the improvements caused by CE in the gut environment.

### Histology of the distal colon

**Fig. 4:**
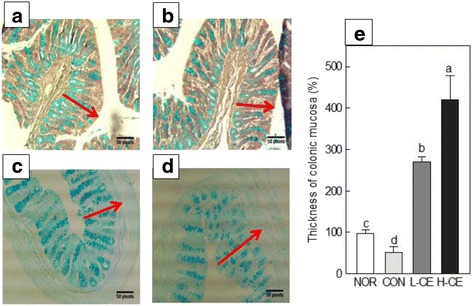
Effects of the CE on histological parameters in the loperamide-induced constipation model. Cross-sections of the distal colon of rats from the NOR (**a**), CON (**b**), L-CE (**c**), and H-CE (**d**) groups. The *arrows* indicate the surface mucus thickness. **e**, Measurement data of the thickness of colonic mucosa. Bars represent the mean ± SD (*n* = 6). Different letters indicate significant differences (*p* < 0.05) among the groups, based on the Tukey’s test. CE, Cheonnyuncho extract; NOR, normal group; CON, constipation control group; L-CE, constipation plus low-dose CE group (3% CE added to drinking water); H-CE, constipation plus high-dose CE group (6% CE added to drinking water)

The results from the observation of intestinal epithelial cells are shown in Fig. 4. Staining of crypt cells showed that mucus secretion in the CON group decreased compared with that in the NOR group but dramatically increased in the L-CE and H-CE groups. These results clearly demonstrated that CE affected the mucus cell activity [24]. The colonic mucosa is covered with a gel layer that protects the underlying epithelium against mechanical damage and chemical irritants in the lumen [25]. Mucin is responsible for the physical and chemical properties of mucus. It has been shown that secretion of colonic mucus decreased in a rat model of spastic constipation, and both thickness of the mucosal layer and the amount of mucin released in the distal colon of rats were decreased by loperamide [26]. Dietary fiber increases the amount of colonic mucus [27]. The mucinase activity in the distal colon is decreased by a high-cellulose diet, and consequently, the amount of colonic mucin may increase.

## Discussion

Cheonnyuncho (*O. humifusa*) is rich in dietary fiber and therefore, the SDF/IDF ratio is as important a nutritional parameter as the TDF content because they cause different physiological effects [20]. SDF usually comprises compounds with high water-holding capacities, which are substrates for intestinal microbiota and contribute to the health status. The CE sample contained 19.45% TDF and 46.12% mucilage (Table 1). The enzyme treatment used for the extraction might have caused hydrolysis of the fiber and decreased the TDF content. The cactus mucilage, which is a polysaccharide complex, is mainly composed of arabinose, galactose, rhamnose, and galacturonic acid [28]. The characteristic sugar composition of the mucilage suggests a strong similarity to pectins, although the former contains lower levels of galacturonic acid [29]. These biological features have all been associated with the prevention of gastrointestinal diseases, colon cancer, and intestinal constipation [30].

In constipation, fecal parameters such as the fecal pellet number and fecal water content have been used as indices of the effect of laxative agents [14]. The results obtained for the fecal pellet numbers and fecal water content showed that the fecal output, measured by the fecal pellet number and water content, was significantly elevated by the administration of CE (Fig. 1). These results indicated that CE decreased constipation in the loperamide-treated animals. We suggest that dietary fiber, one of the main components of the cactus, may account for this effect. Cacti contain dietary fiber, and beneficial effects of fiber have been reported for hypertension, diabetes, obesity, gastrointestinal disease, hyperlipidemia, and immune function [31–33]. Lee and Hwang [34] reported that cellulose increases the fecal excretion by increasing the water content, bulk, and viscosity and that both water-soluble and insoluble cellulose increases the fecal excretion [35]. These previous studies provide the rationale for identifying cellulose as an active component of CE against constipation. Cellulose is a matrix that decreases food digestion by coliform bacteria and increases the fecal water content and bulk [36].

The effect of CE on the intestinal transit ratio may also be explained by the action of the putative active component of CE, cellulose. Soluble fiber has consistently been reported to accelerate peristaltic activity by maintaining higher acidity levels because fiber fermentation results in SCFA production by intestinal coliform bacteria in rats [37]. Generally, the cactus pulp is an important source of pectin, which constitutes up to 70% of its total raw fiber, while the peel and seeds are composed of cellulose, with its levels constituting 71 and 83%, respectively [38]. The intestinal transit ratio increases by activated peristalsis after administration of dietary fiber to rats [39]. Peristalsis and its related physiological/structural changes may be interpreted as the effects of the CE fiber, which induces intestinal transit and prevents constipation.

Our results showed that the triglyceride, total cholesterol, and HDL cholesterol levels were not significantly different among the L-CE, H-CE, and CON groups, which is not unusual. A recent study has reported that pectin reduces cholesterol absorption [40]. According to the report by Ghaffarzadegan et al. [41], cholesterol and triglyceride levels were affected by pectin, which reduced triglycerides and plasma and liver cholesterol. However, the reduction ratios of triglycerides and cholesterol were significantly influenced by the degree of pectin esterification. Low-methoxylation pectin reduced cholesterol and triglyceride levels in fat-diet–fed rats, unlike high-methoxylation pectin. Thus, the source and type of pectin may affect blood cholesterol.

Our results showed that the contents of acetic, propionic, butyric, and valeric acids increased in the fecal samples. This factor influences the composition and metabolic activity of colonic microbiota. The availability of a particular dietary fiber is important for the formation of SCFAs, with different types of dietary fiber giving rise to different SCFA amounts and profiles during colonic fermentation. An increased amount of SCFAs in the intestine is a suitable environment for the growth of probiotic microorganisms [40, 42, 43]. The formation of SCFAs helps improve regularity and laxation by increasing the fecal weight and bulk and fecal water-holding capacity [44]. Our findings indicated that CE was fermented by colonic bacteria and might have increased the number of beneficial bacteria, simultaneously alleviating constipation and improving the bowel environment. Therefore, it is believed that CE could be used as a good prebiotic.

The observation of intestinal epithelial cells showed that CE affected the mucus cell activity. Dietary fiber increases the amount of colonic mucus [27]. The mucilage activity in the distal colon is decreased by a high-cellulose diet, and the amount of colonic mucin may increase. Thus, the supply of dietary fiber may increase mucus release into the lumen and decrease mucus degradation in the colonic lumen. According to our data, the thickness of the epithelial tissue layer and the density of mucin-containing crypt cells in the rat distal colon decreased upon loperamide treatment in the CON group but recovered appreciably in the CE diet groups.

## Conclusion

Our results demonstrate that loperamide-induced constipation was alleviated by the administration of CE to rats. The CE-treated rats showed improved fecal parameters such as the number of fecal pellets, fecal water content, gastrointestinal transit, and SCFA content. Histopathologic evaluation revealed that the supplementation with CE increased the mucus content in epithelial cells of the loperamide-treated rats. Thus, treatment with CE improves constipation through changes to the intestinal environment, including the intactness of the intestinal epithelium and therapy of a disturbed mucosal barrier. Our results demonstrate that Cheonnyuncho (*O. humifusa*) alleviates the symptoms of loperamide-induced constipation.

## References

[1] Andromanakos N, Skandalakis P, Troupis T, Filippou D (2006). Constipation of anorectal outlet obstruction: Pathophysiology, evaluation and management. J Gastroenterol Hepatol.

[2] Sanabria T (2013). Five part herbal cleanse protocol (detoxifying tea, tonifying tea, relaxing tea, parasite tea, intestinal tea) in conjunction with yoga and a vegan alkalinizing food and juice cleanse program. Google Patents.

[3] Heaton KW, Radvan J, Cripps H, Mountford RA, Braddon FEM, Hughes AO (1992). Defecation Frequency and Timing, and Stool Form in the General-Population–a Prospective-Study. Gut.

[4] Sonnenberg A, Koch TR (1989). Epidemiology of constipation in the United States. Dis Colon Rectum.

[5] Lee NYJC, Byub MW (2005). Application of irradiation technology for development of functional natural materials. Food Ind Nutr.

[6] Patel S (2014). Opuntia cladodes (nopal): Emerging functional food and dietary supplement. Mediterr J Nutr Metab.

[7] Hahm S-W, Park J, Son Y-S. Opuntia humifusa stems lower blood glucose and cholesterol levels in streptozotocin-induced diabetic rats. Nutr Res. 2011;31:479–87. 10.1016/j.nutres.2011.05.002 21745630

[8] Cho JY, Park SC, Kim TW, Kim KS, Song JC, Kim SK, Lee HM, Sung HJ, Park HJ, Song YB (2006). Radical scavenging and anti-inflammatory activity of extracts from Opuntia humifusa Raf. J Pharm Pharmacol.

[9] Feugang JM, Konarski P, Zou D, Stintzing FC, Zou C. Nutritional and medicinal use of Cactus pear (Opuntia spp.) cladodes and fruits. Front Biosci. 2006;11:2574–89. 10.2741/1992 16720335

[10] Lee KSOC, Lee KY (2005). Antioxidative efffect of the fractions extracted from a Cactus Cheonnyuncho (Opuntia humifusa). Korean J Food Sci Technol.

[11] Cho IK, Seo KS, Kim YD (2009). Antimicrobial Activities, Antioxidant Effects, and Total Polyphenol Contents of Extracts of Prickly Pear, Opuntia ficus indica. Korean J Food Preserv.

[12] Kim JH, Lee HJ, Lee H-S, Lim E-J, Imm J-Y, Suh HJ (2012). Physical and sensory characteristics of fibre-enriched sponge cakes made with Opuntia humifusa. LWT Food Sci Technol.

[13] Prosky L, Asp NG, Schweizer TF, Devries JW, Furda I (1988). Determination of Insoluble, Soluble, and Total Dietary Fiber in Foods and Food-Products–Interlaboratory Study. J Assoc Off Anal Chem.

[14] Wintola OA, Sunmonu TO, Afolayan AJ (2010). The effect of Aloe ferox Mill. in the treatment of loperamide-induced constipation in Wistar rats. BMC Gastroenterol.

[15] Yu LL, Liao JF, Chen CF (2000). Anti-diarrheal effect of water extract of Evodiae fructus in mice. Ethnopharmacol.

[16] Demigne CRC (1985). Stimulation of absorption of volatile fatty-acids and minerals in the cecum of rats adapted to a very high-fiber diet. Br J Nutr.

[17] Ayadi MA, Abdelmaksoud W, Ennouri M, Attia H (2009). Cladodes from Opuntia ficus indica as a source of dietary fiber: Effect on dough characteristics and cake making. Ind Crop Prod.

[18] Majdoub H, Picton L, Le Cerf D, Roudesli S (2010). Water Retention Capacity of Polysaccharides from Prickly Pear Nopals of Opuntia Ficus Indica and Opuntia Litoralis : Physical–Chemical Approach. J Polym Environ.

[19] Brownlee LA, Havler ME, Dettmar PW, Allen A, Pearson JP (2003). Colonic mucus: secretion and turnover in relation to dietary fiber intake. Proc Nutr Soc.

[20] Leung FW (2007). Etiologic factors of chronic constipation: review of the scientific evidence. Dig Dis Sci.

[21] Yoon WB, Hong YK, Jun HI, Cha MN, Kim YS (2014). Flow behaviors of fruit and stem extracts from Korean cactus (Opuntia humifusa). Food Sci Biotechnol.

[22] Jung EYYS, Suh HJ (2014). Hypocholesterol Effect of Opuntia humifusa Extract on High Cholesterol Diet-induced Hypercholesterolemic Rats. J Korean Soc Food Sci Nutr.

[23] Hu JL, Nie SP, Xie MY (2013). High pressure homogenization increases antioxidant capacity and short-chain fatty acid yield of polysaccharide from seeds of Plantago asiatica L. Food Chem.

[24] Osuna-Martínez U, Reyes-Esparza J, Rodríguez-Fragoso L (2014). Cactus (Opuntia ficus-indica): a review on its antioxidants properties and potential pharmacological use in chronic diseases. Nat Prod Chem Res.

[25] Burger-van Paassen N, Vincent A, Puiman P, van der Sluis M, Bouma J, Boehm G, van Goudoever H, Van Seuningen I, Renes IB (2009). The Regulation of the Intestinal Mucin MUC2 Expression By Short Chain Fatty Acids: Implications for Epithelial Protection. Biochem J.

[26] Shiau SY, Ong YO (1992). Effects of Cellulose, Agar and Their Mixture on Colonic Mucin Degradation in Rats. J Nutr Sci Vitaminol.

[27] Satchithanandam S, Klurfeld DM, Calvert RJ, Cassidy MM (1996). Effects of dietary fibers on gastrointestinal mucin in rats. Nutr Res.

[28] Lee S-P, Whang K, Ha Y-D (1998). Functional properties of mucilage and pigment extracted from Opuntia ficus-indica. J Korean Soc Food Sci Nutr.

[29] Stintzing FC, Schieber A, Carle R (2001). Phytochemical and nutritional significance of cactus pear. Eur Food Res Technol.

[30] Weickert MO, Pfeiffer AF (2008). Metabolic effects of dietary fiber consumption and prevention of diabetes. J Nutr.

[31] Whelton SP, Hyre AD, Pedersen B, Yi Y, Whelton PK, He J (2005). Effect of dietary fiber intake on blood pressure: a meta-analysis of randomized, controlled clinical trials. J Hypertens.

[32] Petruzziello L, Iacopini F, Bulajic M, Shah S, Costamagna G (2006). Review article: uncomplicated diverticular disease of the colon. Aliment Pharmcol Ther.

[33] Watzl B, Girrbach S, Roller M (2005). Inulin, oligofructose and immunomodulation. Brit J Nutr.

[34] Lee HJHE (1997). Effects of alginic acid, cellulose and pectin level on bowel function in Rats. Korean J Nutr.

[35] Lupton JR, Morin JL, Robinson MC (1993). Barley Bran Flour Accelerates Gastrointestinal Transit-Time. J Am Diet Assoc.

[36] Trepel F (2004). Dietary fibre: More than a matter of dietetics. I. Compounds, properties, physiological effects. Wien Klin Wochenschr.

[37] Fuller S, Beck E, Salman H, Tapsell L (2016). New Horizons for the Study of Dietary Fiber and Health: A Review. Plant Food Hum Nutr.

[38] El Kossori RL, Villaume C, El Boustani E, Sauvaire Y, Mejean L (1998). Composition of pulp, skin and seeds of prickly pears fruit (Opuntia ficus indica sp.). Plant Food Hum Nutr.

[39] Kim MJLS (1995). The effect of dietary fiber on the serum lipid level and bowel function in rats. Korean J Nutr.

[40] Ondarza MA (2016). Cactus Mucilages: Nutritional, Health Benefits and Clinical Trials. J Med Biol Sci Res.

[41] Ghaffarzadegan T, Marungruang N, Fåk F, Nyman M (2016). Molecular properties of guar gum and pectin modify cecal bile acids, microbiota, and plasma lipopolysaccharide-binding protein in rats. PLoS One.

[42] Glenn G, Roberfroid M (1995). Dietary modulation of the human colonic microbiota: introducing the concept of prebiotics. J Nutr.

[43] Gibson G, Macfarlane G, Cummings J (1988). Occurrence of sulphate‐reducing bacteria in human faeces and the relationship of dissimilatory sulphate reduction to methanogenesis in the large gut. J Appl Bacteriol.

[44] Slavin JL, Savarino V, Paredes-Diaz A, Fotopoulos G (2009). A review of the role of soluble fiber in health with specific reference to wheat dextrin. J Int Med Res.

